# Directed evolution: selection of the host organism

**DOI:** 10.5936/csbj.201209012

**Published:** 2012-10-27

**Authors:** Azadeh Pourmir, Tyler W. Johannes

**Affiliations:** a Department of Chemical Engineering, The University of Tulsa, 800 S. Tucker Dr, Tulsa, OK 74104, United States

**Keywords:** directed evolution, host organism, protein engineering, microalgae

## Abstract

Directed evolution has become a well-established tool for improving proteins and biological systems. A critical aspect of directed evolution is the selection of a suitable host organism for achieving functional expression of the target gene. To date, most directed evolution studies have used either *Escherichia coli* or *Saccharomyces cerevisiae* as a host; however, other bacterial and yeast species, as well as mammalian and insect cell lines, have also been successfully used. Recent advances in synthetic biology and genomics have opened the possibility of expanding the use of directed evolution to new host organisms such as microalgae. This review focuses on the different host organisms used in directed evolution and highlights some of the recent directed evolution strategies used in these organisms.

## Introduction

**Figure 1:**
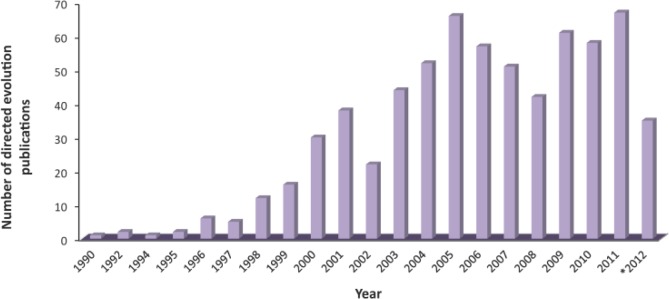
Number of publications per year from 1990 to July 2012 that included the phrase “directed evolution” in the title based on a PubMed database search. * indicates only publications through July 2012.

Directed evolution is a powerful method for improving proteins and other biological molecules and systems, and involves an iterative process of applying selective pressure to a library of variants to identify mutants with desirable properties. Since its development in the early 1990s, directed evolution has become a valuable tool used in protein engineering [1], metabolic engineering [2], biosynthetic pathway engineering [3], and synthetic biology [4, 5]. An analysis of articles published from 1990 to 2012 using the National Institutes of Health PubMed database shows that articles with the phrase “directed evolution” have been published at a steady rate of approximately 50 articles per year since 2004 (Figure 1). The regularity with which these studies appear in the literature emphasizes how effective direction evolution can be at altering and optimizing protein function.

Over the past 20 years, directed evolution has been used successfully to improve protein activity [6], stability [7], substrate specificity [8], enantioselectivity [9], soluble expression [10], and binding affinity [11]. Directed evolution relies on the simple yet powerful Darwinian principles of mutation and selection and is comprised of three essential steps: functional expression of the target protein, generation of DNA diversity, and development of a reliable high-throughput screening assay. Among these steps, selection of a suitable host organism is a prerequisite to library generation and library screening. Selecting an appropriate host organism is critical to achieving functional expression of the target gene; however, actually choosing the best expression system is often challenging and requires the careful consideration of many factors whose potential impacts are hard to predict [12]. Expression of a foreign gene in a non-native host is frequently limited by differences in the expression systems from the native organism. These differences in expression can be caused by a number of factors such as different codon usage, missing chaperones, and posttranslational modifications such as glycosylation or disulfide bridges [13]. Some incompatibilities between the target gene and heterologous host, such as recognition of signal sequences or codon usage, can often be overcome by codon optimization of the target gene sequence [14].

**Figure 2:**
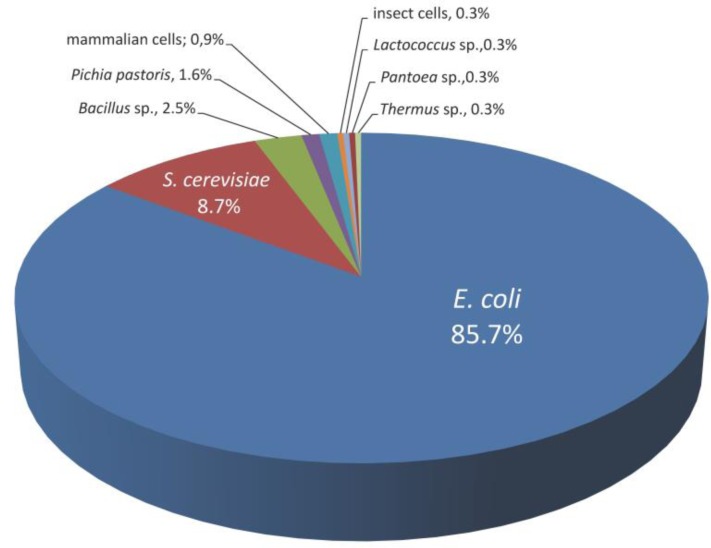
Breakdown of direction evolution studies by host organism. Data is based on a Pubmed database search for articles that included the phrase “directed evolution” in the title.

Although in theory any organism might serve as a host for directed evolution, in reality only a handful have been used. Far and away the most popular host organisms for directed evolution are *Escherichia coli* and *Saccharomyces cerevisiae* because of their high transformation efficiencies, rapid growth rates, well-established manipulation tools, and ability to maintain stable plasmids. To date, *E. coli* has been used in ∼86% of the directed evolution studies published, while *S. cerevisiae* has been used in ∼9% (Figure 2). Other host organisms such as *Bacillus subtilis*, *Bacillus thuringiensis*, *Thermus thermophilus*, *Pantoea agglomerans*, *Lactococcus lactis*, *Pichia pastoris*, mammalian cells (CHO, 3T3, Ramos B-cells), and insect cells (*Spodoptera frugiperda* Sf9) have also been used, but on a more limited basis.

In this review, we discuss the different host organisms used in directed evolution and summarize some recent successful examples for each. A summary of the characteristics and genetic tools available for these organisms is summarized in Table 1. Several other related reviews have been recently published covering synthetic biology [4], biocatalyst development [15], and specifically using *S. cerevisiae* as a host for directed evolution [16].

**Table 1:** Summary of the characteristics and genetic tools available for the host organisms discussed in this review.

Host organism	Doubling time (h)	Transformation efficiency (CFU/µg DNA) (a)	Self-replicating plasmids available?	Protein secretion possible?	Surface display possible?
Bacteria					
*E. coli*	0.25-0.33	10^8^-10^10^	✓	✓	✓
*B. subtilis*	0.50-0.67	10^5^-10^7^	✓	✓	✓
Yeast					
*S. cerevisiae*	1.25-2	10^7^-10^8^	✓	✓	✓
*P. pastoris*	1.5-2	10^5^-10^6^	✓	✓	✓
Mammalian cells					
CHO(b)	14-17	10^7^ (c)	✓	✓	✓
3T3 fibroblasts	18-19	10^7^ (c)	✓	✓	✓
Ramos B-cells	13-14	10^7^ (c)	✓	✓	✓
Insect cells					
*S. frugiperda* Sf9	48-72	10^5^-10^8^	✓	✓	✓
Microalgae					
*Synechocystis* 6803	12-28	10^-5^	✓	✓	✓
*C. reinhardtii* c137	6–8	n = 10^5^ (d), c = 10^-5^ (e)	✓	✓	✓

(a) CFU = colony forming units

(b) CHO = Chinese hamster ovaries

(c) Typical transfection library size

(d) n = nuclear genome

(e) c = chloroplast genome

## Bacteria

### E. coli

Over the past two decades, the Gram-negative bacterium *Escherichia coli* has become the workhorse for most directed evolution studies because of its relative simplicity, well understood genetics, available cloning vectors, collection of mutant host strains, and rapid growth rate [12]. *E. coli* also has a high transformation efficiency (>10^9^ transformants units per µg of plasmid DNA), which is an important factor in preparing large mutant libraries [17]. Significant progress has been made during the last few years regarding the directed evolution of different enzymes in *E. coli*. One particularly impressive example involves the efforts of Arnold and coworkers to use iterative rounds of random mutagenesis, recombination of beneficial mutations, and screening for activity on successively smaller alkanes to convert a cytochrome P450 fatty acid hydroxylase into a propane hydroxylase [18–19]. This approach resulted in a complete respecialization of the P450 BM3 enzyme for a new target substrate by only mutating ∼2% of the amino acid sequence [1]. In another example, the activity of a multi-component aniline dioxygenase enzyme from *Acinetobacter* sp. (AtdA) was enhanced for the bioremediation of a wider range of aromatic amines after one round of saturation mutagenesis followed by error-prone PCR [20]. The engineered biocatalyst from this work seems to hold promise in the remediation of harmful aromatic amine contaminants.

Recently, Jia et al. [21] improved the activity of thermostable β-1,3-1,4-glucanases from *Paecilomyces thermophila* at acidic pH by employing a combined error-prone PCR and DNA-shuffling approach. The optimal pH of the final engineered mutant was shifted from 7 to 5 without any other changes to the enzyme's properties. A new technique has also been developed for screening mutant libraries expressed on the cell surface of *E. coli*. This high-throughput EstA-mediated cell surface display method was used to identify and isolate enantioselective hydrolytic enzymes in *E. coli* [22].

### Bacillus subtilis

*E. coli* is not always the best choice as a host organism, especially when screening enzymes whose substrates cannot be transported across the cell membrane [17]. In this case, an alternative host such as the Gram-positive bacterium *Bacillus subtilis* may be more appropriate. *B. subtilis* has been used as a host for the directed evolution of secretory enzymes such as proteases, lipases, and cellulases [23].

*B. subtilis* has an inherent capacity for secreting a variety of extracellular enzymes directly into the culture medium, which then simplifies downstream purification [24]. In contrast to *E. coli*, *B. subtilis* is considered a generally recognized as safe (GRAS) microorganism and does not produce endotoxins which complicates downstream processing [25]. In addition, *B. subtilis* has other advantages such as an absence of significant codon bias, extensively studied genetics, and well-developed tools for genetic manipulations [26].

Despite its potential application in directed evolution, the use of *B. subtilis* as a host has remained limited. One major drawback is the difficulty in cloning and transforming a mutant library into *B. subtilis* [17]. Direct transformation of *B. subtilis* with a mutant library prepared based on traditional cloning techniques is not efficient. To avoid this, libraries are usually constructed in *E. coli* and then the purified mutant library is transferred into competent *B. subtilis* cells [17]. This method is time-consuming, labor-intensive, and has low efficiency [19]. These difficulties could be avoided if the target enzyme were evolved directly in the *Bacillus* production strain in order to ensure efficient and secreted expression of the target enzyme [23].

Recent progress has been made in addressing some of the challenges. In one study, a simple (restriction enzyme-, phosphatase- and ligase-free), fast (one day), and high-efficiency (∼10^7^ transformants per µg of plasmid DNA) method was developed for directed evolution of a cellulase enzyme using only *B. subtilis* [17]. In another study, Ljubica et al. developed a highly efficient transformation protocol to generate large libraries (∼10^5^ transformants/µg of plasmid DNA) in *B. subtilis* DB104 for the directed evolution of a protease [23]. Recently, the spore coat of *B. subtilis* was used to display a library of laccase enzymes [8]. This spore cell-surface display system was used to identify a mutant laccase (CotA) with 120-fold higher substrate specificity towards the peroxidase substrate ABTS [diammonium 2,2’-azino-bis(3-ethylbenzothiazoline-6-sulfonate].

### Other bacterial species

Besides *E. coli* and *B. subtilis*, the bacterial species *Pantoea agglomerans*, *Lactococcus lactis*, and *Thermus thermophilus* have also been used in directed evolution studies. Zhao and coworkers evolved the nonribosomal peptide synthetase AdmK to generate new derivatives of the antibacterial compound andrimid by targeting mutations to the substrate binding site and generating hundreds of enzymes variants in the native producer, *Pantoea agglomerans* [27]. *Lactococcus lactis* was used as a host for directed evolution of *Listeria monocytogenes* internalin A (InIA) [28]. Random mutagenesis of InIA was combined with cell surface display on *L. lactis* in order to screen novel variants with enhanced infectivity in a murine oral infection model. The extreme thermophile, *Thermus thermophilus*, was used as a host to evolve a mutant kanamycin-resistance enzyme with a 20°C increase in thermostability compared to the wild-type enzyme [29].

## Yeast

### Saccharomyces cerevisiae

*S. cerevisiae* is the most popular host for evolving eukaryotic proteins and enzymes [12]. Recently, Albalde and coworkers published a thorough review based on using *S. cerevisiae* as a host for directed evolution [16], so only a brief overview is provided here. *S. cerevisiae* allows for mutant libraries to be expressed in the cytosol [30], secreted outside the cell [31], or displayed on the cell surface [32]. *S. cerevisiae* also has an efficient DNA recombination apparatus that permits a wide range of genetic manipulations to be employed, thus both homologous recombination and yeast gap repair can be used to rapidly construct and express a library of variants [33]. An especially attractive feature of using *S. cerevisiae* for directed evolution is the increasing number of tools available for generating diversity by assembling different combinations of genetic elements. These tools include the DNA assembler method [34], the COMPACTER method [35], IVOE (*I*n *V*ivo *O*verlap *E*xtension) [36], and the IvAM (*I*n *v*ivo *A*ssembly of *M*utant libraries) approach [37].

*S. cerevisiae* is used routinely as a host in directed evolution and several recent articles have demonstrated its effectiveness. Bulter et al. improved the expression (8-fold) and total activity (170-fold) of a laccase from *Myceliophthora thermophile* in *S. cerevisiae* after nine generations of evolution [12]. In another study, a horseradish peroxidase (HRP) enzyme was engineered in *S. cerevisiae* for enhanced activity [38]. After three rounds of directed evolution by random mutagenesis and screening, a 40-fold increase in total HPR activity was obtained. Recently, a xylose isomerase from *Piromyces* sp. was evolved in *S. cerevisiae* through three rounds of mutagenesis and growth-based screening for improved xylose catabolism and fermentation [39]. A strain expressing the engineered enzyme improved its aerobic growth rate by 61-fold and both ethanol production and xylose consumption rates by 8-fold. The mutant enzyme also enabled ethanol production under oxygen-limited conditions, unlike the wild-type enzyme.

### Pichia pastoris

The use of the methylotrophic yeast, *P. pastoris*, as a host for heterologous production of a variety of eukaryotic proteins has become increasingly popular. *P. pastoris* can be genetically manipulated fairly easily and grown to high cell density in batch culture [40]. Its similarity to *S. cerevisiae* also makes it attractive as a host for directed evolution. *P. pastoris* is a eukaryote and thus has the ability to produce soluble, correctly folded recombinant proteins, either intracellularly or extracellularly with the appropriate post-translational modifications such as glycosylation, disulfide bond formation, and proteolytic processing [41]. For directed evolution studies, a convenient PCR-based technology has been developed that enables efficient library construction and reliable expression through gene integration in *P. pastoris* [42].

Several enzymes have been improved through directed evolution in *P. pastoris* recently. For example, cellobiohydrolase II (CBHII) from the thermophilic fungus *Chaetomium thermophilum* was mutagenized through *in vitro* directed evolution by Wang and coworkers [43]. After screening, two mutants were identified with enhanced CBHII activity. In another example, lipase A from *Candida Antarctica* (CalA) was subjected to directed evolution by the CAST (combinatorial active-site saturation test) method [44]. After multiple rounds of directed evolution, enzyme variants with high enantioselectivity towards both (*R*)-and (*S*)-4-nitrophenyl 2-methylheptanoate were identified. The study also clearly showed the advantages of using the episomal vector pBGP1 in *P. pastoris* for heterologous expression in directed evolution experiments.

## Mammalian cells

Mammalian cells have been employed in directed evolution to engineer recombinant proteins that require posttranslational modifications such as antibodies, hormones and cytokines [45]. Bacteria and yeast are less suitable to evolve these types of proteins because they have insufficient disulfide-bridge formation mechanisms, lack glycosylation, and frequently form protein aggregates [46]. The ability to evolve mammalian proteins within mammalian cells is a more recent development and should decrease the development time for generating, robust high-producing mammalian cells lines for commercial applications [44, 47].

Compared to bacteria and yeast, mammalian cells have low productivity due to their slow growth rates and tendency to undergo programmed cell death (apoptosis) [45]. In addition to these disadvantages, using mammalian cells in directed evolution has also been hampered because the cells are time-consuming to work with, have a low efficiency of stable gene integration, have a tendency toward multiple gene insertions, and display highly variable expression levels [47–48]. Yet despite these difficulties and challenges, mammalian cells have been used successfully as a host for directed evolution. In one study, an anti-apoptosis protein Bcl-x_L_ was evolved by harnessing the somatic hypermutation ability of Ramos B-cells [48]. Mutants of Bcl-x_L_ with high levels of expression were selected and isolated based on survival in the presence of an apoptotic insult. In another study, Chen and coworkers combined error-prone PCR with a high-throughput mammalian cell-surface-tethered screening system in 3T3 fibroblast cells to generate human β-glucuronidase (hβG) variants with enhanced catalytic activities over an extended pH range [49]. Recently, CHO (Chinese hamster ovary) cells were used in a new random lentiviral mutagenesis screening method for the directed evolution of the β_3_ integrin to assess its role in transmembrane topography [50].

## Insect cells

Insect cells are a well known expression system for production of complex proteins. Their popularity stems from their ability to produce relatively large quantities of post-translationally modified eukaryotic proteins in a relatively short amount of time. Insect cells have also been shown to perform most of the same processing steps that occur in mammalian cells [51]. Despite this, the use of insect cells in directed evolution has remained limited largely due to the difficulties in library creation. To date, only one study has been reported that used insect cells as a host for directed evolution. In this study, the human pMHCII (peptide-major histocompatibility complex class II) complex was engineered to improve T cell receptor (TCR) binding affinity [52] in the insect cell line *Spodoptera frugiperda* Sf9. For this study, a system based on insect cell surface display was developed for the functional expression of heterodimeric DR2 molecules with or without a covalently bound human myelin basic protein (MBP) peptide. This insect cell surface display system should aid in efforts to develop new clinical techniques for monitoring the behavior of T cells with improved sensitivity.

## Microalgae

Bacterial, yeast, mammalian, and even insect cell lines have all been used as hosts for directed evolution, but surprisingly no published reports have focused on using microalgae as of yet. Currently, there are intensive global research efforts aimed at increasing or modifying hydrocarbons and other energy storage compounds in microalgae [53]. In the past, a lack of genetic tools and genetic information hampered researcher's ability to engineer enzymes and metabolic pathways in microalgae; however, there now exists a wide array of new genetic manipulation tools, genomic sequences, and high-throughput analytical techniques that should allow scientists to use microalgae as a host for directed evolution studies. Microalgae are often classified into several groups that include diatoms, green algae, golden brown, prymnesiophytes, eustigmatophyes, and cyanobacteria [54]. It should be noted that cyanobacteria are not technically algae but a class of photosynthetic bacteria. This section of the review focuses on evaluating the potential for using cyanobacteria and green algae in directed evolution studies, as these two groups have received the most attention recently for their use in the development and production of algal biofuels and valuable co-products.

### Cyanobacteria

Cyanobacteria, also known as blue-green algae, are photosynthetic bacteria that use light, water, and carbon dioxide to synthesize their energy storage components, i.e. lipids, carbohydrates, and proteins. Cyanobacteria are considered to be a promising feedstock for bioenergy generation based on their lipid accumulation, simple and inexpensive cultivation, and fast growth rates compared to other algae and higher plants [55]. Being prokaryotes, cyanobacteria are also much more amenable to genetic engineering approaches compared to eukaryotic algae. *Synechocystis* sp. PCC 6803 is one of the most widely studied cyanobacteria and serves as a model system for studying photosynthesis, adaptability to environmental stresses, the evolution of plant plastids, and carbon and nitrogen assimilation [56]. This freshwater cyanobacterium can be grown either autotropically or heterotropically (using glucose as a carbon source; however, even though it can grown in complete darkness, for unknown reasons it still requires a small amount of light daily [57]) under a wide range of conditions.

The doubling rate of *Synechocystis* sp. PCC 6803 under optimal conditions is ∼12 hours. *Synechocystis* sp. PCC 6803 has a relatively simple genome and was the first photosynthetic organism to have its entire genome fully sequenced [58]. This strain can efficiently integrate foreign DNA into its genome by homologous recombination and thus allows for targeted gene replacement. Using this feature, a large number of deletion mutants have been created that aid in the study of gene function in cyanobacteria [59]. Extra-chromosomal self-replicating plasmids have been identified for *Synechocystis* sp. PCC 6803 and for the closely related strain *Synechocystis* PCC 6714 [60–61] and an efficient protein secretion method has been developed for *Synechocystis* [62]. There are three widely used gene transfer mechanisms for cyanobacteria: natural transformation [63], conjugation [64], and electroporation [65]. Natural transformation has been shown to have the highest efficiency of the three methods; however, the best efficiency reported for this method (10^-5^ transformants/µg of DNA) is significantly lower than either *E. coli* (10^8^ to 10^10^ transformants/µg of DNA) or *S. cerevisiae* (10^7^ to 10^8^ transformants/µg of DNA) [65–66]. This low transformation efficiency would limit the size of a mutant library and make a directed evolution effort extremely challenging. Another possible limitation is the strong codon bias often observed for the *Synechocystis* genome [67]; however, codon optimization of the target gene has been shown to significantly improve protein expression levels [68], thus codon bias in *Synechocystis* is unlikely to seriously hamper a directed evolution effort.

### Green Algae

Green algae are a large group of algae that share a common ancestry with higher plants. This group of algae has been used extensively in industrial aquaculture, primarily for the production of nutraceuticals, such as omega-3 fatty acids and β-carotene. *Chlamydomonas reinhardtii* is the most widely studied green algae and serves as a model algal organism in the study of photosynthesis, cellular division, flagellar biogenesis, and mitochondrial function [69]. *C. reinhardtii* can be grown either autotrophically or heterotrophically (using acetate as a carbon source) and has a doubling time of 8 hours under optimal conditions. *C. reinhardtii* was the first green algae species to have its nuclear [70], chloroplast [71], and mitochondrial [72] genomes fully sequenced. Transformation methods have been developed that effectively target each of the three genomes [73–75]; however, researchers have mainly focused on transforming the nuclear and chloroplast genomes. Electroporation or agitation with glass beads [76] using a cell-wall deficient strain are the preferred methods used for introducing foreign DNA into the nuclear genome, whereas microparticle bombardment is the standard method for transforming the chloroplast genome [77]. To date, the best transformation efficiency achieved for the nuclear genome is 10^5^ transformants per µg of DNA (by electroporation), whereas only 10^-5^ transformants per µg of DNA has been possible in the chloroplast genome. Both nuclear and chloroplast transformations involve DNA integration into the target genome. Self-replicating plasmids have been identified for *C. reinhardtii* and are likely located in the nuclear compartment of the cell [78]. A protein secretion system and a cell surface display method have been developed for *C. reinhardtii*. Several proteins have been successfully secreted into the cell media by using the export signal sequence of the *Chlamydomonas ARS2* gene in a cell wall deficient *C. reinhardtii* strain [79–80]. Recently it was discovered that the LCl1 protein of *C. reinhardtii*, which is involved with the light-dependent uptake of inorganic carbon, can be used to anchor heterologous proteins to the outer surface of the plasma membrane of *C. reinhardtii* [81–82]. Codon bias has been observed for both the nuclear (high GC%) and chloroplast (high AT%) genomes; however, codon optimization strategies have been successfully used to express high levels of heterologous proteins from both genomes [83–84].

## Conclusion and perspectives

Proper section of the host organism is a critical aspect of directed evolution. Fortunately, a number of bacterial, yeast, insect, and mammalian cell lines are currently available; however, somewhat surprisingly microalgae has yet to be demonstrated as a viable host for directed evolution. It seems likely that in the next few years that both the cyanobacterium *Synechocystis* and the green alga *C. reinhardtii* will be used as host organisms for directed evolution based on their assortment of well-established genetic tools and the widespread interest in algal biofuels and co-products. In order to make this possible for *Synechocystis* however, new methods that improve transformation efficiency will likely be necessary.
